# Cancer-associated fibroblasts are associated with poor prognosis in solid type of lung adenocarcinoma in a machine learning analysis

**DOI:** 10.1038/s41598-021-96344-1

**Published:** 2021-08-18

**Authors:** Kyueng-Whan Min, Dong-Hoon Kim, Yung-Kyun Noh, Byoung Kwan Son, Mi Jung Kwon, Ji-Yong Moon

**Affiliations:** 1 Department of Pathology, Hanyang University Guri Hospital, Hanyang University College of Medicine, Kyoungchun-ro 153, Guri-si, Gyeonggi-do 11923 Republic of Korea; 2 Department of Pathology, Kangbuk Samsung Hospital, Sungkyunkwan University School of Medicine, 29 Saemunanro, Jongno-gu, Seoul, 03181 Republic of Korea; 3 Department of Computer Science, Hanyang University, Seoul, Republic of Korea; 4 School of Computational Sciences, Korea Institute for Advanced Study, Seoul, Republic of Korea; 5 Department of Internal Medicine, Uijeongbu Eulji Medical Center, Eulji University School of Medicine, Gyeonggi-do, Republic of Korea; 6 Department of Pathology, Hallym University Sacred Heart Hospital, Hallym University College of Medicine, Anyang, Gyeonggi-do Republic of Korea; 7 Department of Internal Medicine, Hanyang University Guri Hospital, Hanyang University College of Medicine, Guri, Gyeonggi-do Republic of Korea

**Keywords:** Cancer, Computational biology and bioinformatics, Drug discovery, Systems biology, Biomarkers, Medical research, Oncology, Pathogenesis

## Abstract

Cancer-associated fibroblasts (CAFs) participate in critical processes in the tumor microenvironment, such as extracellular matrix remodeling, reciprocal signaling interactions with cancer cells and crosstalk with infiltrating inflammatory cells. However, the relationships between CAFs and survival are not well known in lung cancer. The aim of this study was to reveal the correlations of CAFs with survival rates, genetic alterations and immune activities. This study reviewed the histological features of 517 patients with lung adenocarcinoma from The Cancer Genome Atlas (TCGA) database. We performed gene set enrichment analysis (GSEA), network-based analysis and survival analysis based on CAFs in four histological types of lung adenocarcinoma: acinar, papillary, micropapillary and solid. We found four hallmark gene sets, the epithelial-mesenchymal transition, angiogenesis, hypoxia, and inflammatory response gene sets, that were associated with the presence of CAFs. CAFs were associated with tumor proliferation, elevated memory CD4+T cells and high CD274 (encoding PD-L1) expression. In the pathway analyses, CAFs were related to blood vessel remodeling, matrix organization, negative regulation of apoptosis and transforming growth factor-β signaling. In the survival analysis of each histological type, CAFs were associated with poor prognosis in the solid type. These results may contribute to the development of therapeutic strategies against lung adenocarcinoma cases in which CAFs are present.

## Introduction

Lung cancer is a major cancer and the most common cause of cancer death in the world^1,2^. According to the National Comprehensive Cancer Network (NCCN) guidelines in oncology, early lung cancer requires surgical procedures, but advanced cancer is treated with systemic treatment^3^. However, about half of the patients recur, usually within the first year after starting treatment^4,5^.

Genetic mutation of cells can induce cancer development, but disease progression and treatment sensitivity are affected by nonmutant cells within the tumor microenvironment^6^. One type of nonmutant cell within the dense collagenous stroma is fibroblast-like cells, so-called cancer-associated fibroblasts (CAFs)^7^. CAFs can drive cancer metastasis through remodeling of the extracellular matrix (ECM) and the production of growth factors and can affect angiogenesis, and these effects influence therapy response^8^. Recently, there has been a growing appreciation of the ability of CAFs to modulate the immune system^6^.

Several studies have reported that survival rates are associated with CAF histological features in different types of malignancy. Previous studies have demonstrated that CAFs and desmoplastic reaction are predictive of poor prognosis in colorectal cancer^9^. Another study suggested that adipocyte-derived fibroblasts are correlated with poor survival and desmoplastic reaction in breast cancer^7^. However, another study reported that histological type, specifically the desmoplastic type, is an independent predictor of favorable prognosis in colorectal cancer^10^.

Recently, molecular studies have utilized bioinformatic tools to find the mechanisms of CAFs. CAFs are a different cell population in terms of origin and pathobiological roles and are derived mainly from mesenchymal stromal cells that are resident in or recruited by the cancer^11^. CAFs are located close to tumor cells and stromal components such as lymphocytes, neutrophils, plasma cells, endothelial cells and ECM^12^. Fibroblasts include CAFs as well as myofibroblastic cells, quiescent fibroblastic cells and pericytic cells. The identification of fibroblasts within the cancer remains challenging due to the lack of specific biomarkers for known and still unclear subtypes^12^.

In recent years, big data analytics and next-generation sequencing (NGS) have allowed the analyses of genetic biomarkers, the quantification of the several types of tumor-infiltrating lymphoid cells and the molecular pathway network-based integration of multiomics data^13–15^. Considering the multiple gene-environment relationships of lung cancer, the clinicopathological application of gene expression data is difficult. For these reasons, we believe that analyses based on gene expression data should focus on identifying a simple, robust, and druggable biomarkers based on high-throughput experimental tools and bioinformatics to achieve accessible therapeutic strategies. The Cancer Genome Atlas (TCGA) has a big database, including digital pathologic slides, clinicopathological information, RNA sequencing, mutation, copy number variable and methylation data^13^. Moreover, the histological features reported in the TCGA database provide data on the presence of CAFs and the tumor microenvironment in lung cancer.

This study aimed to determine whether the presence of CAFs contributes to the clinical outcomes of lung cancer and to evaluate the prognostic value of CAFs^16^. We further aimed to find the gene sets related to CAFs based on gene set enrichment analysis (GSEA)^14^ and molecular pathway network analyses^17,18^. The relationships between lymphoid cells and CAFs were analyzed^15^.

## Materials and methods

### Patient selection

We obtained a total of 1,053 non-small-cell lung carcinoma (NSCLC) cases comprising 566 lung adenocarcinomas and 487 squamous cell carcinomas with known mRNA expression and mutation data from the TCGA database^13^. The analysis was performed on 517 cases containing both virtual histopathological slides and clinical data (from a total of 566 lung adenocarcinoma samples).

### Cancer-associated fibroblasts

**Figure 1:**
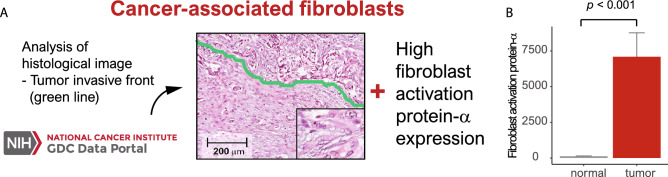
(**A**) Histological images showing cancer-associated fibroblasts at the tumor invasive front (green line) (inset: immature cancer-associated fibroblasts) (original magnification × 400; inset × 1000) and high fibroblast activation protein-α (FAP-α) expression. (**B**) Bar plots showing the difference in fibroblast activation protein-α expression between normal and tumor tissues (*p* < 0.001).

In this review, cells with both immature fibroblastic proliferation (proportion: > 10%) at the tumor invasive front and high FAP gene expression were defined as CAFs (Fig. 1A)^19,20^. To determine the optimal cutoff value for FAP expression, we generated receiver operating characteristic (ROC) curves comparing sensitivity versus 1–specificity. The cutoff value calculated by the ROC curve was used to evaluate the relationship between survival and FAP expression. On the basis of the ROC curve, FAP expression was classified as low (mRNA level ≤ 562.9965) or high (mRNA level > 562.9965). Of the 517 cases, CAFs were present in 101 cases (19.5%).

### Gene set enrichment analysis and pathway-based network analysis based on TCGA data

To detect significant gene sets, GSEA (version 4.1.0) was performed with 31,117 gene sets in the Molecular Signatures Database (MSigDB version 7.2) from the Broad Institute at MIT^14^. Specific gene sets (50 hallmark gene sets) were tested to determine which were associated with CAFs. For this analysis, 1000 permutations were utilized to calculate the *p* values, and the permutation type was set to phenotype. Significant gene sets were those with the following characteristics: false discovery rate < 0.001; family wise-error rate ≤ 0.001; and *p* < 0.001.

We analyzed tumor-infiltrating lymphocytes using deep learning-based lymphocyte classification with convolutional neural networks in whole-slide image analysis and identified immune subtypes using in silico cytometry analyses [CIBERSORT (https://cibersort.stanford.edu/) with Kallisto algorithms]^15,21–23^.

Pathway-based network analysis was based on the identified genes linked to CAFs using Cytoscape (version 3.8.1) network visualization software. To visualize the biological relevance of the histological subtypes and their relevant elements on the basis of GSEA, we performed functional enrichment analyses using ClueGO, an application within Cytoscape software^17,18^.

### Machine learning algorithm for validation

We integrated CAFs with clinical risk factors (T stage, N stage, age, sex, smoking history) to composite prognostic models for survival prediction by applying machine learning (ML) algorithms in 517 cases (randomization: train set, 70%; validation set, 30%). A learning algorithm was independently applied to select and combine multiple covariates from gradient boosting machines (GBM) based on multivariate Bernoulli models. In this step, ‘‘forward” search method, which initiates on a prototype set and selects a feature if and only if the addition of the feature could increase the performance of the prognostic model, is adopted to select optimal features sequentially. Hyperparameters of the ML algorithms, such as learning rate in GBM were optimized for each combination of selected covariates and learning algorithm by grid search cross-validation through a predefined range. We searched across 81 models with varying learning rates and tree depth. The final optimal models were trained based on the selected covariates and the optimized hyperparameters^24^. To explore the performances of the GBM method, the receiver operator characteristic (ROC) curve was used.

### Statistical analysis

Student’s t-test was used to evaluate the differences or relationships among continuous paramters. Disease-free survival (DFS) and disease-specific survival (DSS) were compared using the log rank test. Multivariate analysis was performed to identify independent prognostic markers for DFS and DSS using a Cox multistep regression model. All data were analyzed using R packages. A two-tailed *p* value < 0.05 was considered statistically significant.

## Results

### CAFs were associated with EMT, angiogenesis, hypoxia and inflammatory response

**Figure 2:**
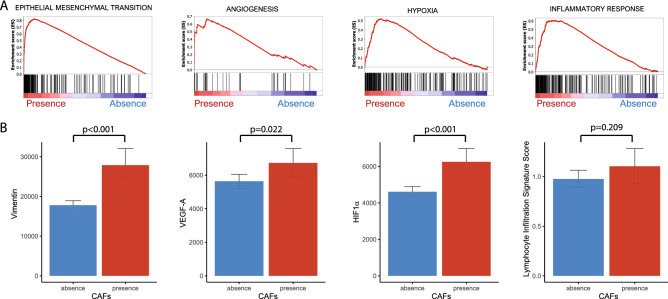
(**A**) Four gene sets (the epithelial-mesenchymal transition, angiogenesis, hypoxia, and inflammatory response gene sets) associated with cancer-associated fibroblasts. (**B**) Bar plots of the relationships between cancer-associated fibroblasts and markers of the identified gene sets: vimentin, vascular endothelial growth factor-A (VEGF-A), hypoxia inducible factor 1 subunit alpha (HIF1α) and lymphocyte infiltration signature score (*p* < 0.001, = 0.022, < 0.001 and = 0.209, respectively).

FAP was highly expressed in tumors compared with normal tissues (*p* < 0.001) (Fig. 1B). We performed GSEA to identify various gene sets associated with CAFs. In the analyses of hallmark gene sets, we found four gene sets (the epithelial-mesenchymal transition, angiogenesis, hypoxia and inflammatory response gene sets) that were associated with lung adenocarcinoma (Fig. 2A).

On the basis of the GSEA results, we analyzed the association between CAFs and each gene set-related marker. Vimentin, a biomarker related to epithelial-mesenchymal transition, was highly expressed in the presence of CAFs (*p* < 0.001). Vascular endothelial growth factor-A (VEGF-A), as a marker related to angiogenesis, was increased in the presence of CAFs (*p* = 0.022). Hypoxia-inducible factor 1 subunit alpha (HIF1α), which is linked to hypoxia, was elevated in the presence of CAFs (*p* < 0.001). The lymphocyte infiltration signature score, which is associated with the inflammatory response, showed a tendency to increase in the presence of CAFs, but it was not statistically significant (*p* = 0.209) (Fig. 2B).

### CAFs were related to low B cells, high CD4+T cells, high PD-L1 expression and proliferation

In the analyses of CAFs, we referred to the immune cell profiles, tumor cell proliferation parameters and biomarkers used in a study by Thorsson et al. and in silico cytometry.^22^

**Figure 3:**
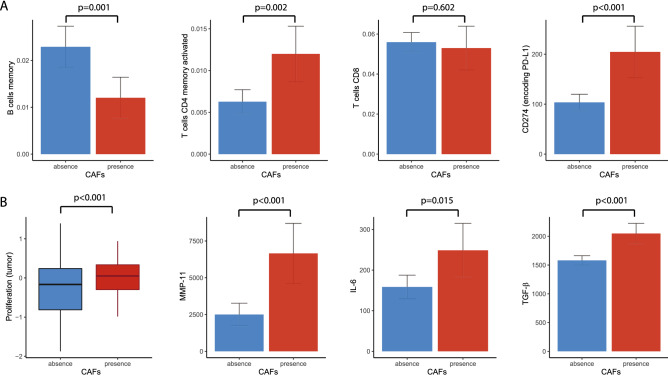
Bar plots of the relationships between cancer-associated fibroblasts and immune factors. (**A**) Memory B cells, activated CD4 + memory T cells, CD8 T cells and CD274 (encoding PD-L1) + cells (*p* = 0.001, 0.002, 0.602 and < 0.001, respectively). (**B**) Relationships between tumor proliferation, matrix metalloproteinase-11 (MMP-11), interleukin-6 (IL-6) and transforming growth factor-β (TGF-β) (*p* < 0.001, 0.001, = 0.015 and < 0.001, respectively).

In comparing the immune cell fractions between samples with and without CAFs, memory B cells were decreased in samples with CAFs (*p* < 0.001), while activated memory CD4+T cells were increased in samples with CAFs (*p* = 0.002). CD274 (encoding PD-L1, programmed death-ligand 1) expression was more elevated in samples with CAFs than in those without CAFs (*p* < 0.001). CD8+T cells showed a tendency to be decreased in samples with CAFs, but this trend was not statistically significant (*p* = 0.602) (Fig. 3A).

The presence of CAFs was associated with higher proliferation and matrix metallopeptidase-11 (MMP-11), interleukin-6 (IL-6) and transforming growth factor-β (TGF-β) levels than the absence of CAFs (*p* < 0.001, < 0.001, = 0.015 and < 0.001, respectively) (Fig. 3B).

### CAFs were linked to blood vessel remodeling, fibrosis and tissue remodeling pathway

**Figure 4:**
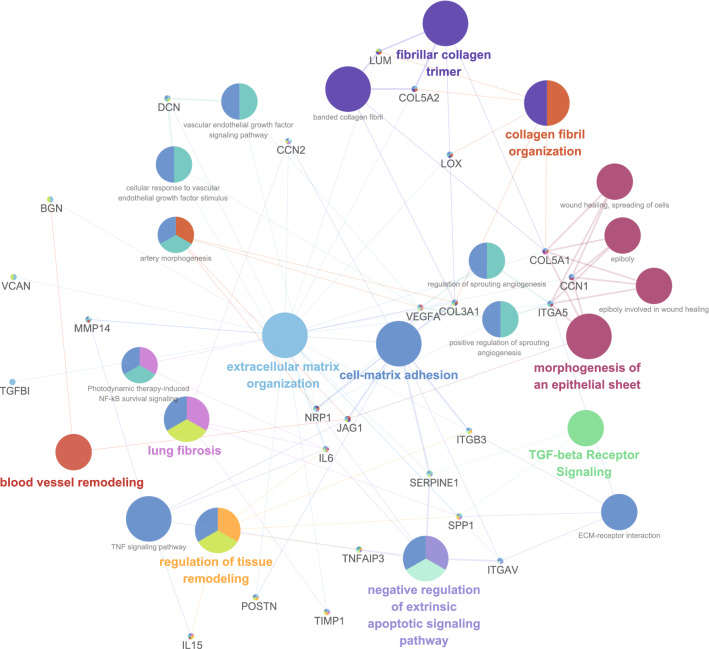
Grouping of networks based on functionally enriched Gene Ontology (GO) terms and pathways associated with the presence of cancer-associated fibroblasts.

We performed pathway-based network analysis using the genes and gene sets associated with CAFs. The CAFs were linked to 10 functionally enriched Gene Ontology (GO) terms and pathways: (1) blood vessel remodeling; (2) lung fibrosis; (3) regulation of tissue remodeling; (4) extracellular matrix organization; (5) cell–matrix adhesion; (6) fibrillar collagen trimer; (7) negative regulation of extrinsic apoptosis signaling pathway; (8) collagen fibril organization; (9) morphogenesis of an epithelial sheet; and (10) TGF-β receptor signaling (Fig. 4).

### CAFS improved survival prediction using machine learning.

In the TCGA data, the distributions of the five predominant histological types were as follows: 7 lepidic type (1.4%), 115 acinar type (22.2%), 107 papillary type (20.7%), 65 micropapillary type (12.6%) and 223 solid type (43.1%). There was an absence of CAFs in the lepidic cases; thus, they were not included in the survival analyses.

**Figure 5:**
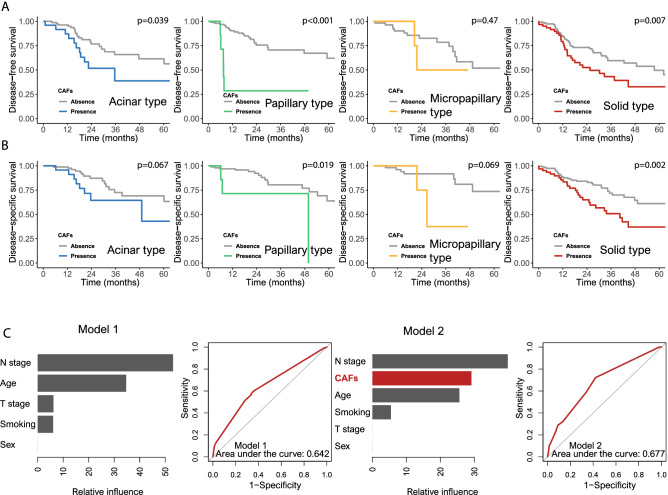
Survival analyses of the four types based on the absence or presence of cancer-associated fibroblasts. (**A**) Disease-free survival: acinar, papillary, micropapillary and solid types (*p* = 0.039, < 0.001, = 0.47 and 0.007, respectively). (**B**) Disease-specific survival: acinar, papillary, micropapillary and solid types (*p* = 0.067, 0.019, 0.069 and 0.002, respectively). (**C**) We supervised machine-learning models for prognosis prediction using gradient boosting machine (GBM). Covariates were included in the confounding factors [Model 1 (left); T stage, N stage, age, sex, smoking (pack years) versus Model 2 (right); Cancer associated fibroblasts (CAFs), T stage, N stage, age, sex, smoking (pack years)] and their relative importance using overall survival. Receiver operator characteristic curve for GBM was used based on a multivariate Gaussian model.

The presence of CAFs was associated with unfavorable DFS and DSS in the acinar type (*p* = 0.039 and 0.067, respectively), but the relationship between CAFs and DSS was not statistically significant. The presence of CAFs was significantly related to shorter DFS and DSS than the absence of CAFs in the papillary type (*p* < 0.001 and 0.019, respectively). The presence of CAFs correlated with worse DFS and DSS in the micropapillary type (*p* = 0.47 and 0.069, respectively), but the correlations were not statistically significant. In the solid type, the presence of CAFs was significantly associated with shorter DFS and DSS than the absence of CAFs (*p* = 0.007 and 0.002, respectively) (Fig. 5A,B). After adjustment for confounders including T stage, N stage, age, sex and smoking history, the presence of CAFs was associated with worse DFS in the papillary type and solid type than the absence of CAFs (*p* < 0.001 and = 0.008, respectively). There was a relationship between shorter DSS and the presence of CAFs in only solid type samples (*p* = 0.003) (Table 1).

**Table 1:** Univariate and multivariate analyses of disease-free survival and disease-specific survival based on cancer-associated fibroblasts.

Covariate	Disease-free survival				Disease-specific survival			
	HR	95%CI		*P* value	HR	95%CI		*P* value
**Acinar type**								
Univariate	2.125	1.021	4.427	0.039	2.202	0.926	5.235	0.067
Multivariate*	1.712	0.775	3.785	0.184	1.842	0.698	4.864	0.217
**Papillary type**								
Univariate	5.460	2.031	14.676	< 0.001	4.061	1.142	14.441	0.019
Multivariate*	13.630	4.112	45.174	< 0.001	3.585	0.706	18.214	0.124
**Micropapillary type**								
Univariate	1.724	0.387	7.677	0.47	3.975	0.796	19.849	0.069
Multivariate*	0.535	0.051	5.642	0.603	3.844	0.367	40.251	0.261
**Solid type**								
Univariate	1.852	1.173	2.925	0.007	2.161	1.296	3.603	0.002
Multivariate*	1.980	1.199	3.269	0.008	2.339	1.328	4.119	0.003

*Adjusted for T stage, N stage, age, sex and smoking history.

We compared the performance of the two GBM models in predicting survival rates (Model 1; T stage, N stage, age, sex, smoking history versus Model 2; CAFs, T stage, N stage, age, sex, smoking history) (Supplementary information and Fig. 1). ROC curves were performed (area under the curve: Model 1, 0.642; Model 2, 0.677) (Fig. 5C). We found that the GBM algorithm performed the best while the addition of CAFs to the prediction model improved the prognostic performance. With cross-validation estimates, 7 decision trees were utilized sequentially while the maximum depth of each decision tree was optimized at 1, corresponding to one-way interactions, and the learning rate was optimized at 0.018.

## Discussion

This study showed survival differences between patients with and without CAFs and analyzed genetic/molecular alterations in patients with lung adenocarcinoma. In previous studies, genetic/molecular signatures related to CAFs have been shown to correlate with prognosis in colorectal, ovarian and breast cancer^25–27^. Our results revealed that the presence of CAFs was associated with a shorter survival rate than the absence of CAFs in lung adenocarcinoma, especially the solid type. In this study, the machine learning model analysis which includes CAFs increased the accuracy of predicting the survival rate. A study by Marcela et al. reported that CAFs were related to increased survival in patients with diffuse large B cell lymphoma^28^. Moreover, another study of colorectal carcinoma demonstrated that the presence of desmoplasia and CAFs was associated with better survival than the absence of desmoplasia^10^. Thus, there is controversy regarding the association between CAFs and the survival of patients with cancer. It is thought that CAFs in the tumor microenvironment are phenotypically heterogeneous and may exhibit both a protumorigenic and antitumorigenic phenotypes^29^. We analyzed hallmark gene sets related to CAFs in lung adenocarcinoma. A total of four gene sets associated with the presence of CAFs were identified: the epithelial-mesenchymal transition (EMT), angiogenesis, hypoxia, and inflammatory response gene sets. Subsequently, we determined the correlations of representative biomarkers associated with these gene sets with to the presence or absence of CAFs. First, vimentin is an EMT biomarker and is involved in cell migration, motility and adhesion and associated with metastasis^30^. Second, VEGF-A is an angiogenesis biomarker and induces high microvascular density and permeability and promotes tumor expansion^31^. Third, HIF1α is a hypoxia biomarker and is associated with the upregulation of glycolytic genes related to oxygen deprivation with increased cancer metabolism^32^. Fourth, the lymphocyte infiltration signature score, which is an inflammatory response marker, is related to prognosis and host-tumor immune interactions in different types of malignancies^33^. Some representative markers, such as vimentin, VEGF-A and HIF1α, were elevated in the presence of CAFs compared to the absence of CAFs. There was no significant difference in the lymphocyte infiltration signature score between samples with and without CAFs. These results suggest that the presence or absence of CAFs has minimal effect on the host-tumor immune response in lung adenocarcinoma.

In addition, we analyzed the changes in immune cell subtypes according to the presence or absence of CAFs by considering the characteristics of different immune cells. Memory B cells were decreased in the presence of CAFs, but activated memory CD4+T cells were increased in the presence of CAFs. CD274 was elevated in the presence of CAFs. There was no significant difference in CD8+T cells between samples with or without CAFs. A study by Costa et al. demonstrated that FAP-high fibroblasts, such as CAFs, are correlated with T_reg_ cell-mediated immunosuppression and poor outcome in breast cancer^34^. Our results showed that, in lung adenocarcinoma, CAFs have little effect on the immunomodulation associated with CD8+T cells in the tumor microenvironment.

CAFs can induce increased levels of growth factors, matrix remodeling and increased levels of numerous cytokines related to immunomodulation^6^. In our results, the proliferation index was increased in the presence of CAFs. TGF-β and IL-6 are related to tumor growth and/or immunosuppression and were increased in the presence of CAFs. A representative marker of ECM and cancer invasion, MMP-11, was elevated in the presence of CAFs. The pathway-based network analysis showed biological functions related to CAFs, such as blood vessel remodeling, extracellular matrix organization, negative regulation of the extrinsic apoptotic signaling pathway and TGF-β receptor signaling.

This study has several limitations that should be acknowledged. First, because this is a cross-sectional study and the in silico analyses with TCGA did not show sustained relationships over time, it is difficult to reach a definitive conclusion. Second, experimental data allowing for novel biological insights into CAFs were not obtained in our study. Further in vitro and/or in vivo studies may be necessary to clarify the molecular mechanisms of CAFs in solid lung adenocarcinoma. Third, CAF function may be highly heterogeneous in solid lung adenocarcinoma patients, as many components of signaling pathways are affected by disease status, microenvironment, and immunity. Fourth, the difficulty in identifying CAFs results largely from the lack of unique markers^6^. In our study, CAFs were defined by a combination of the histological features of fibroblasts and high FAP gene expression. Fifth, we used machine learning and general statistical methods to predict survival differences between patient with or without CAFs. Because CAFs were identified as important factors in predicting survival in both methods, our study based on limited data could not explain the difference between machine learning, which focuses on prediction, and statistical analysis, which focuses on inference. Discussion of the issues will require future research.

This study demonstrated that CAFs are associated with increased tumor cell growth, angiogenesis, and ECM remodeling, effects that produce an unfavorable prognosis in patients with solid lung adenocarcinoma. CAFs were found to be associated with enhanced recruitment of activated memory CD4+T cells with high CD274 expression. The presence of CAFs was related to decreased numbers of CD8+T cells, but the relationship was not statistically significant. Patients with CAFs with high CD274 expression without elevated CD8+T cells might develop resistance to anti-PD-L1 therapies. Our workflow results regarding CAFs will contribute to designing future clinical and experimental studies for patients with solid lung adenocarcinoma.

## Supplementary Information

Supplementary Information.

## Data Availability

The authors declare that all data supporting the findings of this study are available within the article.

## Abbreviations

CAFs: Cancer-associated fibroblasts
ECM: Extracellular matrix
TCGA: The Cancer Genome Atlas
GSEA: Gene set enrichment analysis
FAP: Fibroblast activation protein-α
GBM: Gradient Boosting Machine
NSCLC: Non-small-cell lung carcinoma
ROC: Receiver operating characteristic
ML: Machine Learning
MSigDB: Molecular signatures database
DFS: Disease-free survival
DSS: Disease-specific survival
VEGF-A: Vascular endothelial growth factor-A
HIF1α: Hypoxia-inducible factor 1 subunit alpha
MMP-11: Matrix metallopeptidase-11
IL-6: Interleukin-6
TGF-β: Transforming growth factor-β
GO: Gene ontology
BRAF: V-raf murine sarcoma viral oncogene homolog B
