# Supplementary material for: Cancer-associated fibroblasts are associated with poor prognosis in solid type of lung adenocarcinoma in a machine learning analysis
Source: Sci Rep. 2021 Aug 18;11:16779. doi: 10.1038/s41598-021-96344-1 (PMC8373913; doi:10.1038/s41598-021-96344-1)
Supplement: Supplementary file 1 — Supplementary Information. [file 41598_2021_96344_MOESM1_ESM.pdf]

# Supplementary information

## R code for machine learning

## Prototypes

```
DF1<-“Raw data file”

set.seed(123)

DF1_gbm <- gbm(   formula = DSS1 ~ T + N + Sex + Age + Smoking + CAFs, data = DF1, var.monotone = NULL, n.trees = 100, interaction.depth = 3, n.minobsinnode = 10, shrinkage = 0.1,     bag.fraction = 0.5, train.fraction = 0.7, cv.folds = 1000, keep.data = TRUE, verbose = TRUE, n.cores = NULL)
```

## Optimization using hyperparameter tuning (grid search)

```
hyper_grid <- expand.grid(shrinkage = c(.01, .1, .3), interaction.depth = c(1, 3, 5), n.minobsinnode = c(5, 10, 15), bag.fraction = c(.65, .8, 1), optimal_trees = 0, min_RMSE = 0)

random_DF1_index <- sample(1:nrow(train_DF1), nrow(train_DF1))
random_DF1_train <- train_DF1[random_DF1_index, ]

for(i in 1:nrow(hyper_grid)) {

  # reproducibility
  set.seed(123)

  # train model
  gbm.tune <- gbm(
  formula = DSS1 ~ T + N + Sex + Age + Smoking + CAFs,
    distribution = "bernoulli",
    data = random_DF1_train,
    n.trees = 5000,
    interaction.depth = hyper_grid$interaction.depth[i],
```

```
        shrinkage = hyper_grid$shrinkage[i],
        n.minobsinnode = hyper_grid$n.minobsinnode[i],
        bag.fraction = hyper_grid$bag.fraction[i],
        train.fraction = .7,
        n.cores = NULL, # will use all cores by default
        verbose = FALSE
    )

    # add min training error and trees to grid
    hyper_grid$optimal_trees[i] <- which.min(gbm.tune$valid.error)
    hyper_grid$min_RMSE[i] <- sqrt(min(gbm.tune$valid.error))
}

hyper_grid %>%
    dplyr::arrange(min_RMSE) %>%
    head(10)
```

## Result for optimization (#1)

| no | Shrinkage | Interaction.depth | N.minobsinnode | Bag.fraction | Optimal_trees | min_RMSE |
|---|---|---|---|---|---|---|
| 1 | 0.3 | 1 | 5 | 0.65 | 4 | 1.038039 |
| 2 | 0.3 | 1 | 7 | 0.65 | 4 | 1.038039 |
| 3 | 0.3 | 1 | 10 | 0.65 | 4 | 1.038039 |
| 4 | 0.3 | 3 | 10 | 0.65 | 3 | 1.041651 |
| 5 | 0.1 | 1 | 10 | 0.8 | 9 | 1.042162 |
| 6 | 0.3 | 3 | 10 | 0.8 | 2 | 1.042983 |
| 7 | 0.3 | 3 | 7 | 0.8 | 2 | 1.043104 |
| 8 | 0.1 | 3 | 7 | 0.65 | 4 | 1.044415 |
| 9 | 0.01 | 1 | 10 | 0.65 | 55 | 1.044445 |
| 10 | 0.01 | 1 | 7 | 0.65 | 55 | 1.044482 |

Shrinkage, learning rate; Interaction.depth, maximum nodes per tree; N.minobsinnode, the minimum number of observations in trees' terminal nodes; Bag.fraction, subsampling fraction; RMSD, root mean square deviation

```
hyper_grid <- expand.grid(shrinkage = c(0.1, .2, .3), interaction.depth = c(1), n.minobsinnode = c(5, 10, 15), bag.fraction = c(.65, .8, 1), optimal_trees = 0, min_RMSE = 0 )

nrow(hyper_grid)

# grid search
```

```
for(i in 1:nrow(hyper_grid)) {

  # reproducibility
  set.seed(123)

  # train model
  gbm.tune <- gbm(
  formula = DSS1 ~ T + N + Sex + Age + Smoking + CAFs,
    distribution = "bernoulli",
    data = DF1,
    n.trees = 5000,
    interaction.depth = hyper_grid$interaction.depth[i],
    shrinkage = hyper_grid$shrinkage[i],
    n.minobsinnode = hyper_grid$n.minobsinnode[i],
    bag.fraction = hyper_grid$bag.fraction[i],
    train.fraction = .7,
    n.cores = NULL, # will use all cores by default
    verbose = FALSE
  )

  # add min training error and trees to grid
  hyper_grid$optimal_trees[i] <- which.min(gbm.tune$valid.error)
  hyper_grid$min_RMSE[i] <- sqrt(min(gbm.tune$valid.error))
}

hyper_grid %>%
  dplyr::arrange(min_RMSE) %>%
  head(10)
```

### Result for optimization (#2)

| no | Shrinkage | Interaction.depth | N.minobsinnode | Bag.fraction | Optimal_trees | min_RMSE |
|---|---|---|---|---|---|---|
| 1 | 0.3 | 1 | 5 | 0.80 | 7 | 0.9053389 |
| 2 | 0.1 | 1 | 5 | 0.65 | 54 | 0.9065168 |

| | | | | | | |
|---|---|---|---|---|---|---|
| 3 | 0.3 | 1 | 10 | 0.80 | 7 | 0.9074224 |
| 4 | 0.3 | 1 | 10 | 0.65 | 7 | 0.9080574 |
| 5 | 0.1 | 1 | 10 | 0.65 | 26 | 0.9081091 |
| 6 | 0.3 | 1 | 5 | 0.65 | 7 | 0.9082037 |
| 7 | 0.1 | 1 | 5 | 1 | 61 | 0.9082157 |
| 8 | 0.1 | 1 | 10 | 0.80 | 31 | 0.9082353 |
| 9 | 0.2 | 1 | 5 | 1 | 23 | 0.9083679 |
| 10 | 0.2 | 1 | 5 | 0.80 | 26 | 0.9085773 |
| Shrinkage, learning rate; Interaction.depth, maximum nodes per tree; N.minobsinnode, the minimum number of observations in trees' terminal nodes; Bag.fraction, subsampling fraction; RMSD, root mean square deviation | | | | | | |

## Optimized code

set.seed(123)

DF1_fit_gbm <- gbm(

formula = DSS1 ~ T + N + Sex + Age + Smoking + CAFs,

data = DF1, var.monotone = NULL, n.trees = 7, interaction.depth =1, n.minobsinnode = 5, shrinkage = 0.3, bag.fraction = 0.8, train.fraction = 0.7, cv.folds = 1000, keep.data = TRUE, verbose = TRUE, n.cores = NULL)

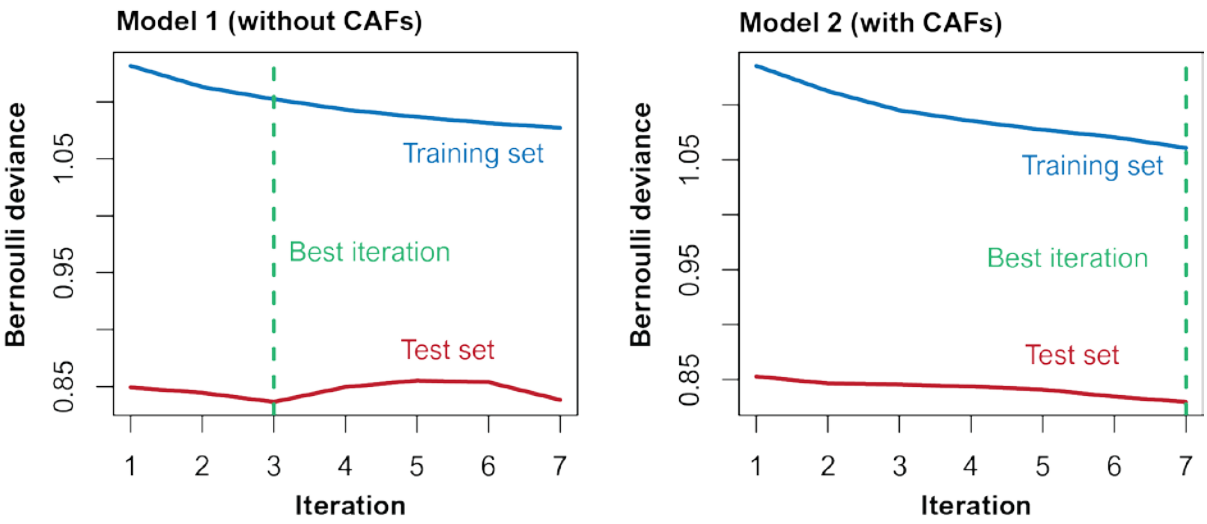


## “Raw data file” for machine learning

| T | Sex | CAFs | DSS1 | N | Age | Smoking |
|---|---|---|---|---|---|---|
| 2 | 0 | 0 | 0 | 2 | 70 | 38 |

| | | | | | | |
|---|---|---|---|---|---|---|
| 2 | 0 | 0 | 0 | 0 | 67 | 52 |
| 3 | 1 | 1 | 0 | 1 | 79 | 47 |
| 2 | 0 | 1 | 0 | 0 | 68 | 62 |
| 2 | 0 | 0 | 0 | 2 | 66 | 20 |
| 1 | 0 | 0 | 0 | 0 | 70 | 43 |
| 2 | 1 | 1 | 0 | 0 | 58 | 15 |
| 4 | 0 | 0 | 0 | 2 | 76 | 0 |
| 4 | 0 | 0 | 0 | 1 | 76 | 19 |
| 2 | 0 | 0 | 0 | 1 | 65 | 45 |
| 4 | 1 | 1 | 0 | 3 | 47 | 72 |
| 2 | 1 | 0 | 0 | #NULL! | 57 | 0 |
| 2 | 0 | 0 | 0 | 0 | 76 | 0 |
| 2 | 1 | 0 | 0 | 0 | 74 | 65 |
| 2 | 0 | 0 | 0 | 0 | 62 | 98 |
| 4 | 0 | 0 | 1 | 2 | 57 | 29 |
| 2 | 1 | 1 | 0 | 0 | 51 | 56 |
| 3 | 0 | 1 | 0 | 2 | 69 | 25 |
| 2 | 0 | 0 | 0 | 0 | 41 | 38 |
| 2 | 0 | 0 | 0 | 0 | 68 | 147 |
| 3 | 0 | 0 | 0 | 0 | 70 | 50 |
| 2 | 1 | 1 | 0 | 0 | 70 | 28 |
| 2 | 0 | 0 | 0 | 0 | 71 | 20 |
| 2 | 1 | 1 | 0 | 1 | 65 | 8 |
| 2 | 1 | 0 | 0 | 0 | 59 | 80 |
| 2 | 0 | 0 | 0 | 1 | 66 | 58 |
| 2 | 0 | 0 | 0 | 0 | 82 | 1 |
| 4 | 1 | 0 | 0 | 1 | 67 | 15 |
| 2 | 0 | 0 | 0 | 2 | 67 | 40 |
| 2 | 0 | 0 | 0 | 1 | 65 | 72 |
| 2 | 0 | 0 | 0 | 1 | 68 | 40 |
| 1 | 0 | 0 | 0 | 1 | 57 | 30 |
| 3 | 0 | 0 | 0 | 2 | 60 | 0 |
| 2 | 1 | 0 | 0 | 0 | 69 | 0 |
| 2 | 0 | 0 | 0 | 0 | 57 | 0 |
| 1 | 0 | 1 | 0 | 0 | 69 | 45 |
| 1 | 0 | 1 | 0 | 0 | 38 | 20 |
| 2 | 0 | 1 | 0 | 2 | 61 | 35 |
| 2 | 1 | 0 | 0 | 0 | 66 | 50 |
| 2 | 1 | 0 | 0 | 0 | 57 | 40 |

| | | | | | | |
|---|---|---|---|---|---|---|
| 1 | 1 | 0 | 0 | 1 | 64 | 0 |
| 2 | 1 | 1 | 1 | 1 | 65 | 0 |
| 3 | 0 | 1 | 1 | 0 | 68 | 100 |
| 2 | 1 | 0 | 1 | 0 | 75 | 0 |
| 2 | 1 | 0 | 1 | 0 | 72 | 40 |
| 2 | 0 | 1 | 1 | 1 | 42 | 10 |
| 2 | 1 | 1 | 0 | 2 | 70 | 0 |
| 1 | 1 | 0 | 1 | 0 | 72 | 25 |
| 2 | 0 | 0 | 0 | 0 | 80 | 12 |
| 1 | 1 | 0 | 0 | 0 | 65 | 30 |
| 2 | 0 | 1 | 0 | 0 | 59 | 23 |
| 2 | 1 | 0 | 0 | #NULL! | 74 | 25 |
| 1 | 1 | 1 | 0 | 1 | 65 | 57 |
| 1 | 1 | 1 | 0 | 0 | 69 | 0 |
| 2 | 0 | 1 | 0 | 0 | 65 | 56 |
| 2 | 1 | 1 | 0 | 1 | 55 | 0 |
| 2 | 0 | 0 | 1 | 0 | 43 | 30 |
| 2 | 0 | 1 | 1 | 0 | 51 | 60 |
| 2 | 1 | 1 | 0 | 2 | 74 | 50 |
| 1 | 1 | 1 | 0 | 0 | 77 | 60 |
| 1 | 1 | 1 | 0 | 0 | 60 | 20 |
| 1 | 1 | 1 | 1 | 0 | 71 | 0 |
| 2 | 1 | 1 | 1 | 0 | 60 | 30 |
| 2 | 0 | 0 | 0 | 2 | 53 | 20 |
| 2 | 1 | 0 | 0 | 0 | 51 | 17.5 |
| 1 | 1 | 0 | 0 | #NULL! | 61 | 5 |
| 1 | 1 | 1 | 0 | 0 | 62 | 75 |
| 3 | 0 | 0 | 0 | 0 | 64 | 15 |
| 1 | 1 | 0 | 0 | #NULL! | 67 | 0 |
| 1 | 0 | 0 | 0 | 0 | 60 | 60 |
| 1 | 1 | 1 | 0 | 2 | 56 | 20 |
| 2 | 1 | 1 | 0 | 0 | 72 | 25 |
| 1 | 1 | 0 | 0 | 0 | 60 | 45 |
| 2 | 1 | 1 | 0 | #NULL! | 85 | 64 |
| 1 | 0 | 0 | 0 | 0 | 59 | 15 |
| 2 | 1 | 1 | 1 | 1 | 50 | 15 |
| 1 | 0 | 0 | 0 | 0 | 70 | 40 |
| 2 | 0 | 0 | 0 | 0 | 72 | 124 |
| 2 | 1 | 1 | 1 | 0 | 69 | 22 |

| | | | | | | |
|---|---|---|---|---|---|---|
| 2 | 0 | 1 | 0 | 0 | 61 | 50 |
| 3 | 1 | 0 | 0 | 0 | 49 | 50 |
| 1 | 0 | 0 | 1 | 1 | 59 | 20 |
| 1 | 1 | 0 | 0 | 1 | 47 | 30 |
| 2 | 0 | 0 | 0 | 0 | 64 | 0 |
| 1 | 1 | 1 | 0 | 0 | 52 | 20 |
| 2 | 1 | 0 | 0 | 0 | 54 | 54 |
| 3 | 0 | 0 | 0 | 0 | 73 | 50 |
| 2 | 0 | 1 | 0 | 0 | 58 | 30 |
| 2 | 1 | 1 | 0 | 0 | 73 | 40 |
| 2 | 1 | 0 | 0 | 0 | 78 | 80 |
| 2 | 0 | 0 | 0 | 0 | 79 | 4.5 |
| 1 | 1 | 0 | 0 | 0 | 73 | 28 |
| 1 | 0 | 1 | 0 | 0 | 73 | 90 |
| 1 | 1 | 0 | 1 | 0 | 67 | 50 |
| 1 | 0 | 0 | 1 | 0 | 72 | 17.5 |
| 1 | 1 | 0 | 1 | 0 | 72 | 40 |
| 1 | 1 | 0 | 0 | 0 | 74 | 0 |
| 3 | 1 | 1 | 1 | 2 | 45 | 5 |
| 3 | 0 | 0 | 1 | 2 | 77 | 50 |
| 2 | 1 | 0 | 1 | 0 | 67 | 0 |
| 2 | 1 | 0 | 1 | 1 | 61 | 74 |
| 2 | 1 | 0 | 0 | 1 | 68 | 100 |
| 3 | 1 | 0 | 1 | 1 | 73 | 78 |
| 2 | 1 | 0 | 1 | 1 | 51 | 55.5 |
| 2 | 1 | 0 | 1 | 2 | 69 | 0 |
| 1 | 1 | 0 | 0 | 0 | 79 | 65 |
| 2 | 0 | 0 | 1 | 1 | 70 | 10 |
| 1 | 1 | 0 | 0 | 2 | 81 | 50 |
| 2 | 1 | 0 | 0 | 1 | 64 | 20 |
| 2 | 0 | 0 | 0 | 2 | 82 | 20 |
| 1 | 1 | 0 | 0 | 2 | 68 | 50 |
| 3 | 1 | 1 | 0 | 0 | 46 | 30 |
| 1 | 1 | 0 | 0 | 1 | 63 | 0 |
| 1 | 0 | 0 | 0 | 0 | 57 | 37 |
| 2 | 0 | 0 | 0 | 0 | 64 | 0 |
| 2 | 0 | 0 | 0 | 1 | 69 | 0 |
| 2 | 0 | 0 | 1 | 2 | 51 | 35 |
| 3 | 0 | 0 | 1 | 0 | 61 | 0 |

| | | | | | | |
|---|---|---|---|---|---|---|
| 1 | 1 | 1 | 1 | 0 | 51 | 15 |
| 1 | 1 | 0 | 0 | 0 | 56 | 0 |
| 1 | 1 | 0 | 0 | 0 | 39 | 0 |
| 2 | 1 | 0 | 0 | 0 | 41 | 60 |
| 1 | 0 | 0 | 0 | 0 | 68 | 0 |
| 1 | 1 | 0 | 1 | 0 | 52 | 30 |
| 4 | 1 | 1 | 1 | 1 | 72 | 0 |
| 2 | 1 | 1 | 1 | 1 | 57 | 0 |
| 2 | 0 | 0 | 0 | 0 | 70 | 0 |
| 2 | 1 | 0 | 1 | 2 | 42 | 30 |
| 1 | 1 | 0 | 1 | 1 | 79 | 0 |
| 2 | 0 | 0 | 0 | 0 | 72 | 0 |
| 2 | 0 | 0 | 0 | 0 | 72 | 0 |
| 2 | 1 | 0 | 1 | 1 | 59 | 0 |
| 2 | 0 | 0 | 1 | 2 | 74 | 15 |
| 2 | 0 | 0 | 1 | 2 | 47 | 0 |
| 2 | 1 | 1 | 1 | 0 | 75 | 40 |
| 2 | 0 | 0 | 1 | 1 | 75 | 0 |
| 4 | 0 | 0 | 0 | 2 | 72 | 0 |
| 1 | 1 | 0 | 0 | 0 | 86 | 0 |
| 2 | 0 | 0 | 1 | 2 | 58 | 0 |
| 2 | 0 | 0 | 0 | 0 | 85 | 15 |
| 2 | 1 | 0 | 0 | 2 | 55 | 25 |
| 1 | 1 | 0 | 0 | 0 | 67 | 30 |
| 1 | 1 | 0 | 0 | 0 | 69 | 0 |
| 1 | 0 | 0 | 0 | 0 | 62 | 0 |
| 1 | 0 | 0 | 0 | 0 | 62 | 0 |
| 2 | 1 | 0 | 0 | 0 | 72 | 50 |
| 2 | 1 | 0 | 1 | 0 | 63 | 0 |
| 2 | 1 | 1 | 1 | 0 | 71 | 0 |
| 1 | 1 | 1 | 1 | 2 | 49 | 0 |
| 3 | 1 | 1 | 1 | 2 | 79 | 0 |
| 2 | 1 | 1 | 1 | 2 | 74 | 0 |
| 2 | 1 | 0 | 0 | 0 | 79 | 0 |
| 1 | 1 | 0 | 0 | 0 | 84 | 0 |
| 1 | 0 | 1 | 1 | 0 | 60 | 120 |
| 1 | 1 | 0 | 0 | 0 | 63 | 30 |
| 3 | 0 | 0 | 0 | 0 | 68 | 10 |
| 1 | 0 | 0 | 0 | 0 | 74 | 0 |

| | | | | | | |
|---|---|---|---|---|---|---|
| 2 | 1 | 0 | 1 | 0 | 40 | 50 |
| 1 | 1 | 0 | 1 | 1 | 76 | 35 |
| 4 | 1 | 0 | 0 | 0 | 51 | 35 |
| 2 | 0 | 0 | 0 | 1 | 63 | 40 |
| 2 | 0 | 0 | 1 | 0 | 65 | 0 |
| 2 | 0 | 0 | 0 | 2 | 68 | 0 |
| 1 | 1 | 0 | 0 | 0 | 74 | 0 |
| 2 | 0 | 0 | 0 | 1 | 55 | 50 |
| 1 | 0 | 0 | 0 | 1 | 58 | 0 |
| 1 | 1 | 0 | 0 | 0 | 60 | 60 |
| 2 | 0 | 0 | 0 | 0 | 63 | 0 |
| 2 | 0 | 0 | 1 | 1 | 71 | 0 |
| 1 | 0 | 0 | 1 | 0 | 61 | 0 |
| 2 | 0 | 0 | 0 | 0 | 52 | 0 |
| 2 | 1 | 0 | 1 | 2 | 67 | 0 |
| 2 | 1 | 0 | 0 | 0 | 59 | 60 |
| 2 | 0 | 0 | 0 | 0 | 72 | 0 |
| 2 | 0 | 1 | 1 | 1 | 61 | 0 |
| 2 | 0 | 1 | 1 | 0 | 81 | 0 |
| 2 | 1 | 1 | 1 | 1 | 59 | 0 |
| 1 | 0 | 0 | 0 | 0 | 56 | 0 |
| 1 | 1 | 0 | 0 | 2 | 53 | 0 |
| 2 | 1 | 1 | 1 | 1 | 79 | 0 |
| 2 | 0 | 0 | 0 | 1 | 81 | 60 |
| 2 | 1 | 0 | 1 | 1 | 71 | 0 |
| 2 | 1 | 0 | 0 | 0 | 58 | 50 |
| 2 | 1 | 0 | 0 | 0 | 74 | 0 |
| 1 | 0 | 0 | 0 | 0 | 77 | 0 |
| 3 | 0 | 0 | 1 | 1 | 77 | 0 |
| 1 | 1 | 0 | 0 | 0 | 70 | 20 |
| 3 | 1 | 0 | 0 | 2 | 76 | 5 |
| 3 | 0 | 0 | 1 | 0 | 74 | 0 |
| 1 | 0 | 0 | 0 | 0 | 60 | 0 |
| 1 | 1 | 0 | 0 | 0 | 72 | 0 |
| 2 | 1 | 0 | 1 | 0 | 64 | 2 |
| 2 | 0 | 0 | 0 | 0 | 54 | 0 |
| 2 | 1 | 0 | 0 | 0 | 76 | 50 |
| 1 | 1 | 0 | 0 | 0 | 68 | 16 |
| 1 | 1 | 0 | 0 | 0 | 72 | 30 |

| | | | | | | |
|---|---|---|---|---|---|---|
| 1 | 0 | 0 | 0 | 2 | 70 | 0 |
| 2 | 1 | 0 | 0 | 0 | 64 | 30 |
| 2 | 0 | 0 | 0 | 0 | 76 | 0 |
| #NULL! | 1 | 1 | 1 | #NULL! | 49 | 0 |
| 1 | 0 | 0 | 0 | 0 | 64 | 40 |
| 2 | 0 | 0 | 1 | 1 | 77 | 80 |
| 2 | 1 | 0 | 0 | 0 | 50 | 15 |
| 1 | 1 | 0 | 0 | 0 | 70 | 96 |
| 1 | 1 | 0 | 1 | 0 | 61 | 40 |
| 1 | 1 | 1 | 1 | 1 | 71 | 5 |
| 3 | 0 | 0 | 0 | 0 | 81 | 28 |
| 1 | 1 | 0 | 0 | 0 | 73 | 60 |
| 1 | 0 | 0 | 0 | 0 | 64 | 0 |
| 2 | 1 | 0 | 0 | 0 | 59 | 0 |
| 1 | 0 | 0 | 0 | 0 | 56 | 97 |
| 1 | 0 | 1 | 1 | 0 | 80 | 0 |
| 2 | 0 | 0 | 0 | 0 | 74 | 0 |
| 3 | 0 | 0 | 1 | 0 | 75 | 40 |
| 2 | 0 | 0 | 0 | 0 | 51 | 70 |
| 2 | 1 | 1 | 1 | 0 | 67 | 104 |
| 1 | 1 | 0 | 0 | 0 | 60 | 35 |
| 1 | 1 | 0 | 0 | 0 | 69 | 50 |
| 2 | 1 | 0 | 0 | 0 | 87 | 0 |
| 2 | 1 | 0 | 0 | 0 | 76 | 30 |
| 1 | 0 | 0 | 0 | 0 | 56 | 0 |
| 2 | 0 | 0 | 0 | 0 | 73 | 0 |
| 1 | 1 | 0 | 0 | 0 | 73 | 50 |
| 1 | 1 | 1 | 1 | 0 | 61 | 20 |
| 2 | 0 | 0 | 0 | 0 | 58 | 100 |
| 2 | 0 | 0 | 0 | 0 | 54 | 59 |
| 1 | 0 | 0 | 0 | 2 | 62 | 0 |
| 3 | 1 | 0 | 0 | 0 | 62 | 0 |
| 1 | 0 | 0 | 0 | 0 | 53 | 120 |
| 2 | 1 | 0 | 0 | 1 | 60 | 0 |
| 2 | 1 | 0 | 0 | 0 | 55 | 0 |
| 2 | 1 | 0 | 0 | 0 | 73 | 58 |
| 1 | 0 | 0 | 1 | 1 | 41 | 19 |
| 3 | 1 | 0 | 0 | 0 | 77 | 0 |
| 2 | 1 | 0 | 0 | 0 | 70 | 20 |

| | | | | | | |
|---|---|---|---|---|---|---|
| 2 | 0 | 0 | 0 | 0 | 76 | 40 |
| 3 | 0 | 0 | 0 | 2 | 67 | 67 |
| 2 | 1 | 0 | 0 | 0 | 58 | 0 |
| 3 | 1 | 0 | 0 | 0 | 72 | 0 |
| 1 | 0 | 0 | 1 | 1 | 60 | 154 |
| 1 | 1 | 0 | 0 | 0 | 75 | 30 |
| 1 | 1 | 0 | 0 | 1 | 63 | 36 |
| 2 | 0 | 0 | 0 | 0 | 69 | 50 |
| 1 | 1 | 0 | 0 | 3 | 60 | 40 |
| 2 | 0 | 0 | 0 | 0 | 78 | 34 |
| 1 | 1 | 0 | 0 | 0 | 81 | 45 |
| 1 | 1 | 0 | 0 | 0 | 70 | 35 |
| 2 | 1 | 0 | 0 | 0 | 54 | 53 |
| 2 | 1 | 0 | 0 | 0 | 61 | 7 |
| 1 | 0 | 0 | 1 | 0 | 88 | 60 |
| 1 | 0 | 0 | 0 | 0 | 71 | 100 |
| 1 | 1 | 0 | 0 | 0 | 80 | 0 |
| 4 | 1 | 0 | 0 | 2 | 65 | 0 |
| 3 | 1 | 0 | 0 | 0 | 80 | 0 |
| 3 | 1 | 0 | 0 | 0 | 70 | 0 |
| 2 | 0 | 0 | 0 | 2 | 55 | 20 |
| 2 | 0 | 0 | 0 | 2 | 62 | 70 |
| 2 | 1 | 0 | 1 | 2 | 73 | 0 |
| 2 | 1 | 0 | 1 | 0 | 65 | 48 |
| 2 | 0 | 0 | 0 | 0 | 65 | 40 |
| 2 | 1 | 0 | 0 | 0 | 54 | 25 |
| 2 | 0 | 0 | 1 | 0 | 73 | 40 |
| 2 | 1 | 0 | 0 | 1 | 71 | 0 |
| 2 | 1 | 0 | 0 | 0 | 78 | 0 |
| 2 | 1 | 0 | 1 | 2 | 70 | 0 |
| 2 | 0 | 0 | 1 | 0 | 84 | 30 |
| 2 | 0 | 0 | 0 | 1 | 64 | 30 |
| 3 | 0 | 0 | 0 | 0 | 70 | 60 |
| 1 | 0 | 0 | 0 | 0 | 58 | 60 |
| 2 | 1 | 0 | 0 | 2 | 77 | 50 |
| 2 | 1 | 0 | 0 | 0 | 70 | 20 |
| 1 | 1 | 0 | 0 | 2 | 58 | 40 |
| 2 | 0 | 0 | 0 | 2 | 63 | 50 |
| 1 | 1 | 0 | 0 | 0 | 61 | 0 |

| | | | | | | |
|---|---|---|---|---|---|---|
| 2 | 0 | 0 | 0 | 0 | 60 | 45 |
| 4 | 0 | 1 | 1 | 0 | 71 | 110 |
| 2 | 0 | 0 | 0 | 0 | 60 | 68 |
| 2 | 0 | 0 | 0 | 2 | 61 | 40 |
| 2 | 1 | 0 | 0 | 0 | 55 | 25 |
| 2 | 0 | 0 | 0 | 1 | 74 | 30 |
| 1 | 1 | 0 | 0 | 0 | 70 | 0 |
| 1 | 1 | 0 | 0 | 0 | 77 | 0 |
| 2 | 1 | 0 | 0 | 0 | 82 | 0 |
| 2 | 1 | 0 | 0 | 0 | 84 | 0 |
| 2 | 1 | 0 | 0 | 0 | 73 | 0 |
| 3 | 0 | 0 | 0 | 0 | 69 | 0 |
| 2 | 1 | 0 | 0 | 0 | 52 | 0 |
| 1 | 1 | 0 | 0 | 0 | 57 | 0 |
| 2 | 1 | 0 | 0 | 1 | 73 | 0 |
| 3 | 0 | 0 | 0 | 0 | 73 | 0 |
| 2 | 0 | 0 | 0 | 0 | 84 | 20 |
| 1 | 0 | 0 | 0 | 0 | 69 | 7.5 |
| 1 | 0 | 0 | 0 | 0 | 75 | 30 |
| 4 | 0 | 0 | 0 | 0 | 56 | 0 |
| 2 | 1 | 0 | 0 | 0 | 42 | 25 |
| 2 | 1 | 0 | 0 | 2 | 54 | 30 |
| 2 | 0 | 0 | 0 | 1 | 59 | 80 |
| 2 | 1 | 0 | 0 | 0 | 71 | 28 |
| 1 | 1 | 0 | 0 | 0 | 70 | 80 |
| 1 | 1 | 0 | 0 | 1 | 59 | 7.8 |
| 2 | 0 | 0 | 0 | #NULL! | 85 | 40 |
| 1 | 0 | 0 | 0 | 0 | 71 | 40 |
| 3 | 0 | 0 | 0 | 0 | 77 | 12 |
| 3 | 1 | 0 | 0 | 0 | 60 | 25 |
| 2 | 1 | 0 | 0 | 0 | 48 | 0 |
| 2 | 1 | 1 | 1 | 0 | 60 | 0 |
| 2 | 1 | 1 | 0 | 0 | 80 | 25 |
| 2 | 0 | 0 | 1 | 2 | 66 | 12 |
| 1 | 1 | 0 | 0 | 0 | 65 | 10 |
| 1 | 1 | 0 | 0 | 0 | 52 | 10 |
| 2 | 1 | 1 | 0 | 1 | 66 | 55 |
| 2 | 1 | 1 | 0 | 0 | 69 | 30 |
| 3 | 0 | 0 | 0 | 1 | 59 | 41 |

| | | | | | | |
|---|---|---|---|---|---|---|
| 2 | 0 | 0 | 1 | 1 | 45 | 48 |
| 2 | 0 | 0 | 1 | 0 | 74 | 0 |
| 1 | 1 | 0 | 0 | 0 | 58 | 28 |
| 2 | 1 | 0 | 0 | 0 | 81 | 0 |
| 3 | 0 | 0 | 1 | 0 | 41 | 11 |
| 2 | 0 | 0 | 1 | 0 | #NULL! | 20 |
| 2 | 0 | 0 | 1 | 1 | #NULL! | 25 |
| 3 | 1 | 0 | 0 | 2 | #NULL! | 40 |
| 2 | 0 | 0 | 0 | 0 | #NULL! | 0 |
| 2 | 1 | 0 | 0 | 0 | #NULL! | 0 |
| 2 | 1 | 0 | 0 | 2 | #NULL! | 0 |
| 2 | 1 | 1 | 1 | 0 | #NULL! | 0 |
| 2 | 0 | 0 | 0 | 0 | #NULL! | 20 |
| 2 | 0 | 1 | 0 | 2 | #NULL! | 10 |
| 2 | 1 | 1 | 1 | 0 | #NULL! | 34 |
| 2 | 1 | 1 | 1 | 1 | #NULL! | 0 |
| 2 | 1 | 0 | 1 | 2 | #NULL! | 40 |
| 2 | 0 | 0 | 0 | 0 | #NULL! | 0 |
| 2 | 0 | 1 | 0 | 0 | #NULL! | 50 |
| 3 | 0 | 1 | 0 | 0 | #NULL! | 0 |
| 2 | 1 | 0 | 0 | 0 | #NULL! | 5 |
| 2 | 1 | 0 | 0 | 0 | 62 | 0 |
| 4 | 1 | 0 | 1 | 1 | 52 | 25 |
| 2 | 1 | 0 | 0 | 2 | 71 | 6 |
| 2 | 1 | 0 | 1 | 1 | 67 | 50 |
| 2 | 0 | 0 | 0 | 1 | 71 | 57 |
| 4 | 0 | 0 | 0 | 0 | 71 | 115 |
| 2 | 0 | 1 | 1 | 1 | 59 | 37 |
| 2 | 0 | 0 | 1 | 0 | 65 | 40 |
| 2 | 1 | 0 | 0 | 0 | 65 | 20 |
| 3 | 0 | 0 | 0 | 2 | 72 | 112.5 |
| 2 | 0 | 0 | 0 | 0 | 68 | 48 |
| 4 | 0 | 0 | 0 | 1 | 62 | 84 |
| 4 | 1 | 0 | 1 | 2 | 59 | 14 |
| 1 | 1 | 1 | 0 | #NULL! | 60 | 21 |
| 4 | 0 | 1 | 0 | 2 | 61 | 43.8 |
| 3 | 1 | 0 | 1 | 0 | 69 | 27.8 |
| 1 | 0 | 0 | 0 | 0 | 75 | 12 |
| 2 | 0 | 0 | 0 | 0 | 60 | 41 |

| | | | | | | |
|---|---|---|---|---|---|---|
| 2 | 0 | 0 | 0 | 1 | 84 | 38 |
| 2 | 0 | 0 | 1 | 0 | 77 | 64 |
| 2 | 1 | 0 | 1 | 2 | 53 | 35 |
| 2 | 0 | 0 | 1 | 0 | 45 | 35 |
| 2 | 0 | 0 | 1 | 2 | 69 | 44 |
| 2 | 0 | 0 | 0 | 0 | 72 | 20 |
| 2 | 1 | 0 | 0 | 0 | 75 | 2.2 |
| 2 | 1 | 0 | 0 | 0 | 66 | 0 |
| 2 | 0 | 0 | 0 | 0 | 56 | 60 |
| 2 | 0 | 0 | 1 | 0 | 67 | 94 |
| 1 | 0 | 0 | 0 | 1 | 59 | 45 |
| 3 | 1 | 1 | 0 | 0 | 58 | 45 |
| 1 | 1 | 0 | 0 | 0 | 77 | 10 |
| 2 | 0 | 0 | 1 | 1 | 69 | 11 |
| 2 | 1 | 0 | 0 | 0 | 53 | 45 |
| 2 | 1 | 0 | 0 | 1 | #NULL! | 15 |
| 1 | 1 | 0 | 0 | 0 | #NULL! | 26 |
| 2 | 0 | 0 | 0 | 0 | #NULL! | 60 |
| 1 | 1 | 1 | 0 | 0 | 59 | 69 |
| 2 | 0 | 0 | 1 | 1 | 52 | 0 |
| 1 | 1 | 0 | 0 | 1 | 73 | 52 |
| 2 | 0 | 1 | 0 | 0 | 66 | 0 |
| 2 | 0 | 0 | 1 | 1 | 70 | 50 |
| 2 | 0 | 0 | 0 | 0 | 70 | 0 |
| 1 | 1 | 1 | 1 | 2 | 61 | 43 |
| 1 | 1 | 0 | 0 | 0 | 69 | 0 |
| 2 | 1 | 0 | 0 | 0 | 68 | 8 |
| 2 | 0 | 0 | 0 | 0 | 62 | 0 |
| 2 | 0 | 0 | 0 | 1 | 61 | 9 |
| 2 | 0 | 1 | 0 | 1 | 79 | 60 |
| 4 | 1 | 0 | 0 | 0 | 63 | 36 |
| 2 | 0 | 0 | 0 | 0 | 58 | 40 |
| 1 | 1 | 1 | 0 | 1 | 62 | 40 |
| 2 | 1 | 0 | 0 | 0 | 66 | 0 |
| 1 | 0 | 0 | 0 | 0 | 42 | 0 |
| 2 | 1 | 1 | 0 | 1 | 63 | 0 |
| 2 | 0 | 1 | 0 | 1 | 46 | 0 |
| 2 | 1 | 0 | 0 | 0 | 54 | 0 |
| 1 | 0 | 0 | 0 | #NULL! | 75 | 30 |

| | | | | | | |
|---|---|---|---|---|---|---|
| 2 | 0 | 0 | 0 | 0 | 44 | 20 |
| 3 | 0 | 0 | 0 | 2 | 52 | 36 |
| 2 | 0 | 0 | 0 | 0 | 57 | 0 |
| 1 | 1 | 0 | 0 | 0 | 61 | 0 |
| 1 | 0 | 0 | 0 | 0 | 64 | 47 |
| 2 | 1 | 0 | 0 | 1 | 72 | 0 |
| 3 | 0 | 1 | 0 | 0 | 59 | 0 |
| 2 | 0 | 0 | 0 | 0 | 61 | 54 |
| 2 | 0 | 0 | 0 | 1 | 50 | 37.5 |
| 1 | 1 | 0 | 0 | 0 | 78 | 0 |
| 2 | 0 | 0 | 0 | 0 | 48 | 0 |
| 3 | 0 | 0 | 1 | 0 | 56 | 0 |
| 2 | 1 | 0 | 0 | 0 | 63 | 0 |
| 1 | 1 | 0 | 0 | 2 | 59 | 0 |
| 1 | 0 | 0 | 0 | 0 | 70 | 0 |
| 2 | 0 | 0 | 0 | 0 | 78 | 94.5 |
| 1 | 1 | 0 | 0 | 1 | 65 | 12 |
| 2 | 0 | 0 | 0 | 0 | 66 | 0 |
| 1 | 1 | 0 | 0 | 0 | 81 | 25 |
| 2 | 1 | 0 | 0 | 0 | 52 | 50 |
| 1 | 1 | 0 | 0 | 0 | 59 | 50 |
| 2 | 1 | 0 | 0 | 0 | 62 | 0 |
| 2 | 0 | 1 | 0 | 2 | 59 | 0 |
| 2 | 1 | 0 | 0 | 2 | 75 | 30 |
| 3 | 0 | 0 | 0 | 0 | 62 | 75 |
| 2 | 1 | 0 | 0 | #NULL! | 63 | 0 |
| 1 | 1 | 0 | 0 | 0 | 75 | 0 |
| 1 | 1 | 0 | 0 | 0 | 76 | 90 |
| 2 | 0 | 1 | 0 | 0 | 59 | 30 |
| 1 | 0 | 0 | 0 | 1 | 78 | 0 |
| 1 | 1 | 0 | 0 | 0 | 76 | 11 |
| 1 | 1 | 0 | 0 | 0 | 75 | 42 |
| 2 | 0 | 0 | 0 | 0 | 77 | 20 |
| 2 | 0 | 0 | 0 | 0 | 71 | 40 |
| 1 | 0 | 0 | 0 | 0 | 70 | 50 |
| #NULL! | 0 | 0 | 0 | #NULL! | 64 | 0 |
| 1 | 0 | 0 | 0 | 0 | 49 | 0 |
| 3 | 1 | 0 | 0 | 0 | 54 | 34 |
| 1 | 1 | 0 | 1 | 0 | 63 | 40 |

| | | | | | | |
|---|---|---|---|---|---|---|
| 2 | 0 | 0 | 0 | 1 | 71 | 10 |
| 2 | 0 | 1 | 0 | 1 | 61 | 0 |
| 1 | 0 | 0 | 0 | 0 | 71 | 50 |
| 1 | 0 | 0 | 0 | 0 | 67 | 100 |
| 2 | 1 | 0 | 0 | 0 | 42 | 0.15 |
| 1 | 0 | 1 | 0 | 0 | 72 | 0 |
| 2 | 0 | 0 | 0 | 1 | 67 | 0 |
| 2 | 1 | 0 | 0 | 2 | 74 | 45 |
| 2 | 1 | 0 | 0 | 1 | 62 | 42 |
| 2 | 1 | 0 | 0 | 2 | 66 | 20 |
| 1 | 1 | 0 | 0 | 0 | 76 | 25 |
| 2 | 1 | 0 | 0 | 0 | 67 | 30 |
| 2 | 0 | 0 | 0 | 0 | 70 | 40 |
| 1 | 1 | 0 | 0 | 0 | 58 | 20 |
| 2 | 1 | 0 | 0 | 2 | 83 | 30 |
| 2 | 0 | 0 | 0 | 0 | 65 | 35 |
| 1 | 1 | 0 | 0 | 0 | 76 | 40 |
| 1 | 1 | 0 | 0 | 0 | 72 | 22.5 |
| 2 | 0 | 0 | 0 | 2 | 81 | 65 |
| 2 | 1 | 0 | 0 | 0 | 75 | 20 |
| 2 | 0 | 0 | 0 | 0 | 67 | 40 |
| 2 | 1 | 0 | 0 | 0 | 55 | 0 |
| 3 | 0 | 0 | 0 | 1 | 63 | 40 |
| 2 | 1 | 0 | 0 | 0 | 59 | 0 |
| 1 | 0 | 0 | 0 | 0 | 72 | 60 |
| 2 | 1 | 1 | 0 | 2 | 78 | 0 |
| 1 | 1 | 0 | 0 | 0 | 55 | 0 |
| 2 | 0 | 0 | 0 | 0 | 81 | 120 |
| 2 | 1 | 0 | 0 | 0 | 60 | 34 |
| 1 | 1 | 0 | 0 | 0 | 52 | 3 |
| 1 | 0 | 0 | 0 | 0 | 66 | 100 |
| 1 | 1 | 0 | 0 | 0 | 69 | 37.5 |
| 1 | 0 | 0 | 0 | 0 | 83 | 6 |
| 1 | 1 | 0 | 0 | 0 | 45 | 0 |
| 1 | 0 | 0 | 0 | 0 | 74 | 40 |
| 4 | 1 | 0 | 0 | 0 | 74 | 52 |
| 3 | 1 | 0 | 0 | 2 | 72 | 20 |
| 1 | 1 | 1 | 0 | 0 | 50 | 30 |
| 1 | 0 | 0 | 0 | 0 | 61 | 90 |

| | | | | | | |
|---|---|---|---|---|---|---|
| #NULL! | 1 | 1 | 1 | #NULL! | 74 | 0 |
| 1 | 1 | 0 | 0 | 0 | 70 | 46 |
| 2 | 1 | 0 | 0 | 1 | 65 | 0 |
| 3 | 1 | 0 | 0 | 0 | 69 | 20 |
| 1 | 1 | 1 | 1 | 0 | 61 | 120 |
| 1 | 1 | 0 | 0 | 0 | 77 | 0 |
| 1 | 1 | 1 | 0 | 0 | 66 | 25 |
| 1 | 1 | 0 | 0 | 0 | 48 | 33 |
| 1 | 0 | 0 | 0 | 0 | 67 | 30 |
| 1 | 1 | 1 | 0 | 0 | 63 | 90 |
| 1 | 1 | 1 | 0 | 0 | 60 | 10 |
| 1 | 0 | 0 | 1 | 1 | 75 | 19 |
| 3 | 1 | 0 | 1 | 2 | 40 | 0 |
| 2 | 0 | 1 | 0 | 1 | 56 | 40.5 |
| 2 | 0 | 0 | 0 | 1 | 69 | 93 |
| 2 | 1 | 0 | 0 | 0 | 64 | 14 |
| 2 | 0 | 0 | 0 | 1 | 60 | 0 |
| 1 | 0 | 0 | 0 | 0 | 57 | 0 |
| 1 | 0 | 0 | 0 | 0 | 63 | 0 |
| 2 | 0 | 0 | 0 | 0 | 67 | 110 |
| 2 | 0 | 0 | 0 | 1 | 53 | 15 |
| 2 | 0 | 1 | 0 | 1 | 61 | 40 |
| 2 | 1 | 1 | 0 | 1 | 68 | 50 |
| 1 | 1 | 0 | 0 | 2 | 76 | 50 |
| 2 | 1 | 0 | 0 | 0 | 75 | 50 |
| 2 | 0 | 0 | 0 | 2 | 68 | 25 |
| 2 | 1 | 1 | 1 | 2 | 54 | 24 |
| 1 | 1 | 0 | 1 | 0 | 75 | 55 |
| 1 | 0 | 0 | 0 | 2 | 77 | 125 |
| 2 | 0 | 1 | 0 | 2 | 71 | 20 |
| 2 | 0 | 0 | 1 | 0 | 56 | 40 |
| 2 | 1 | 0 | 1 | 0 | 58 | 40 |
| 1 | 1 | 0 | 0 | 0 | 70 | 20 |
| 2 | 0 | 1 | 1 | 1 | 72 | 35 |
| 1 | 1 | 1 | 0 | 0 | 62 | 0 |
| 2 | 1 | 1 | 1 | 1 | 56 | 28 |
| 2 | 1 | 0 | 0 | 0 | 76 | 15 |
| 1 | 1 | 0 | 0 | 0 | 50 | 30 |
| 2 | 0 | 1 | 0 | 0 | 65 | 104 |

| | | | | | | |
|---|---|---|---|---|---|---|
| 2 | 1 | 0 | 0 | 2 | 87 | 3 |
| 2 | 0 | 1 | 0 | 0 | 52 | 60 |
| 1 | 1 | 0 | 0 | 0 | 69 | 40 |
| 2 | 1 | 0 | 0 | 0 | 76 | 25 |
| 1 | 1 | 0 | 0 | 1 | 56 | 30 |
| 1 | 0 | 0 | 0 | 0 | 67 | 5 |
| 4 | 0 | 0 | 0 | 1 | 49 | 0 |
| 1 | 1 | 0 | 1 | 0 | 74 | 45 |
| 1 | 1 | 0 | 0 | 0 | 68 | 95 |
